# Plasma exchange as potential treatment of severe immune checkpoint inhibitor-induced hepatitis

**DOI:** 10.1016/j.jhepr.2025.101684

**Published:** 2025-11-20

**Authors:** Lucy Meunier, Clement Monet, Antonio Saviano, Marwin Farrugia, François Villeret, Fanny Lebossé, Marion Khaldi, Olivier Moranne, Christine Chambon, Philippe Ichai, Astrid Laurent-Bellue, Ariane Laparra, Rodolphe Anty, Simona Tripon, Mialy Randrianarisoa, Alexandre Maria, Lina Hountondji, Eleonora De Martin

**Affiliations:** 1 Liver Unit, Saint-Eloi Hospital, INSERM 1183, Montpellier School of Medicine, Montpellier, France; 2 Department of Anesthesia and Intensive Care unit, Regional University Hospital of Montpellier, St-Eloi Hospital, University of Montpellier, Montpellier, CEDEX 5, France; PhyMedExp, INSERM U1046, CNRS UMR, 9214, Montpellier, France; 3 Gastroenterology and Hepatology Service, Strasbourg University Hospitals, Strasbourg, France; 4 Archet 2 University Hospital, Inserm U1065 Team 8, Université Côte d'Azur, Nice, France; 5 Department of Hepatology, Hospices Civils de Lyon; Université Claude Bernard Lyon 1; INSERM Unit 1350 PathLiv; Lyon Hepatology Institute, IHU EVEREST; Lyon, France; 6 CHU Nantes, Nantes Université, Service Hépato-Gastroentérologie, IMAD, Nantes, France; 7 Service de Nephrologie-Dialyse-Apherese, Hôpital Universitaire Carémeau Nimes, IDESP Université de Montpellier, France; 8 Department of Hepatology, Hôpital Edouard Herriot, Hospices Civils de Lyon, University of Lyon, 69003, Lyon, France; 9 AP-HP Hôpital Paul-Brousse, Centre Hépato-Biliaire, INSERM Unit 1193, Université Paris-Saclay, FHU Hepatinov, Villejuif, F-94800, France; 10 Hôpital Kremlin Bicêtre, Anatomie & Cytologie Pathologiques, Le Kremlin Bicetre, France; 11 Interdisciplinary Department for the Organization of Patient Pathways-DIOPP, Gustave Roussy, Villejuif, France; 12 Internal Medicine and Multi-Organic Diseases Department, Hôpital Saint Éloi, CHU Montpellier, IRMB, University of Montpellier, INSERM, Montpellier, France

**Keywords:** Therapeutic plasma exchange, Checkpoint inhibitor-induced liver injury, Acute liver failure

## Abstract

**Background & Aims:**

Immune checkpoint inhibitors (ICIs) improve cancer survival but can cause severe immune-related hepatitis. Standard therapy with corticosteroids and second-line agents such as mycophenolate mofetil fails in some cases. Therapeutic plasma exchange (TPE) has been suggested as rescue therapy, but supporting evidence is limited.

**Methods:**

We retrospectively analyzed French multicenter data (March 2021–April 2025) on patients with grade 4 ICI-induced hepatitis refractory to corticosteroids ± other immunosuppressants who underwent TPE.

**Results:**

Thirteen patients (median age 63 years; 54% male) were included. Eleven had acute hepatitis, including seven with acute liver injury, and two had steroid-refractory cholestatic hepatitis. TPE was initiated a median of 28 days after hepatitis onset (median MELD score 21) and administered in 2–8 sessions. Liver function improved in eight patients (61.5%), allowing resumption of anticancer therapy without ICI rechallenge in five. Three patients with cirrhosis and hepatocellular carcinoma died of liver failure, and three died from other causes. TPE-related adverse events were mild to moderate.

**Conclusion:**

TPE is feasible, safe, and may be effective for severe steroid- and immunosuppressant-refractory ICI-induced hepatitis. Early initiation could improve outcomes; prospective studies are needed to clarify optimal timing and patient selection.

**Impact and implications:**

Severe immune checkpoint inhibitor-induced hepatitis represents a rare but life-threatening complication for which therapeutic options are limited once corticosteroids and immunosuppressants fail. Our study provides the first multicenter evidence suggesting that therapeutic plasma exchange (TPE) is a feasible and safe approach that may improve hepatic outcomes in this setting. These findings are particularly relevant for oncologists, hepatologists, and intensivists facing refractory immune checkpoint inhibitor-induced liver injury, as TPE could offer a bridge to recovery when liver transplantation is not feasible. Early consideration of TPE, alongside careful patient selection, could optimize outcomes, although larger prospective studies are required to confirm efficacy and define the best timing and indications.

## Highlights

• This French multicenter cohort is the first systematic evaluation of TPE for severe ICI-induced hepatitis refractory to therapy.
• Liver function improved or normalized in 61.5%, enabling resumption of non-ICI anticancer therapy in several patients.
• Non-responders more often had encephalopathy and higher post-TPE MELD scores, highlighting the importance of early selection.
• TPE may be a rescue option for non-transplant candidates, supporting prospective studies on timing and indications.

## Graphical abstract

**Figure:**
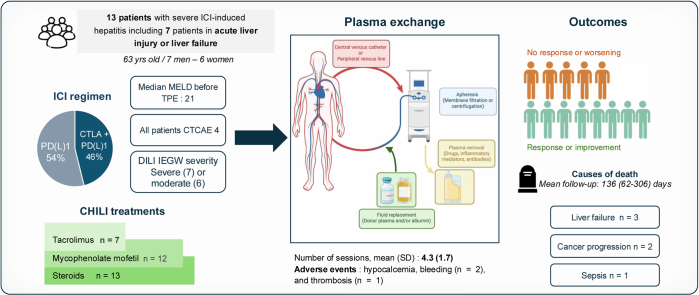

## Introduction

The advent of immune checkpoint inhibitors (ICIs) has significantly transformed the therapeutic management of many cancers, leading to improved survival in a large number of patients. However, by modulating immune tolerance, these agents can also induce immune-related adverse events (irAEs), affecting all organs.^1^ Among these, liver toxicity is a frequent complication, occurring in up to 25% of patients depending on the ICI combinations used.^2^ While most ICI-induced hepatitis cases are asymptomatic to moderate, severe forms, including acute liver injury (ALI), have been described. These may progress to acute liver failure (ALF), a potentially life-threatening condition. Death from ICI-induced hepatitis remains rare, with an estimated incidence of 0.07% to 0.5% .^3^ Data from the VigiLyze database identified 613 fatal ICI-related toxic events reported internationally between 2009 and January 2018, of which 22% were attributed to hepatitis.^4^ Current guidelines recommend initiating treatment with high-dose corticosteroids. In cases of failure, additional immunosuppressive agents such as mycophenolate mofetil are advised.[5], [6], [7] However, some cases prove refractory to these conventional therapies, presenting a major therapeutic challenge.^8^^,^^9^ In other severe immune-related toxicities, particularly cardiac (*e.g*. myocarditis), neurological, or muscular, therapeutic plasma exchange (TPE) has been successfully used.^4^^,^^10^ This extracorporeal technique enables the removal of pathogenic plasma components, including antibodies or therapeutic immunoglobulins such as ICIs, due to their high molecular weight and low extravascular distribution volume. IrAEs have been newly recognized as a potential indication for TPE, although the level of evidence remains low.^8^^,^^9^^,^^11^ Despite this rationale, data on the use of TPE for severe ICI-induced hepatitis remain scarce, consisting mostly of isolated case reports.^12^^,^^13^ The efficacy and safety of this approach in this specific context have not yet been systematically evaluated. Therefore, the aim of this study was to assess, through a national cohort, the feasibility, tolerance, and potential benefit of TPE in patients with severe ICI-induced hepatitis refractory to both corticosteroids and additional immunosuppressive therapy.

## Materials and methods

### Study design and data collection

We conducted a retrospective multicenter national study to identify all cases of immune checkpoint inhibitor-induced liver injury (CHILI) for which the physician used TPE. All French hepatology and intensive care units managing patients with irAEs were contacted. We included all patients who developed hepatitis during treatment with ICIs and underwent at least one session of TPE for hepatic toxicity. For each patient, clinical data were collected, including underlying malignancy, medical history, ICI treatment regimen, diagnosis of hepatic toxicity, therapeutic management, and follow-up. Laboratory data were recorded at baseline, during hepatitis episodes, and before and after each treatment line. TPE characteristics were also collected: number of sessions, infusion route, exchanged volume, and potential complications.

### Definitions^14^

Hepatitis severity was assessed using the CTCAE classification, DILI Network and DILI Expert Working Group criteria, as well as the model for end-stage liver disease (MELD) score. The hepatitis pattern was assessed using the alanine aminotransferase/alkaline phosphatase serum ratio (R value = (alanine aminotransferase/upper limit of normal)/(alkaline phosphatase/upper limit of normal), and classified as cholestatic (R ≤2), hepatocellular (R ≥5), or mixed (2< R <5). Responders were defined as patients who achieved either complete recovery, indicated by normalization of liver enzymes, or partial improvement, defined as a decrease in transaminase levels to below grade 1 according to CTCAE criteria. ALI was defined as jaundice (hyperbilirubinemia ≥42.5 μmol/L) and international normalized ratio ≥1.5; and ALF was defined as ALI with clinical hepatic encephalopathy.

### Management

Decisions regarding specific treatments, including treatment-line changes and dosing, were made at the discretion of the referring physician at each center, based on current guidelines.[5], [6], [7]

### TPE complications

Complications of plasma exchange were defined as any procedure-related adverse events, such as allergic reactions, catheter-related infections or thrombosis, bleeding, and electrolyte imbalances.

### Statistical analysis

Descriptive statistics were used to summarize the study population. Continuous variables were expressed as mean ± SD or median (IQR), depending on data distribution. Categorical variables were presented as frequencies and percentages. Easymedstat software was used for statistical analysis (version 3.30.2; www.easymedstat.com).

The study protocol was approved by the appropriate Institutional Review Board (approval number A060/2025-01-196/001).

## Results

Between March 2021 and April 2025, 13 patients from eight centers were included: seven men and six women, with a median age of 63 years (IQR 57–67) (Table 1). Most patients were treated for stage IV cancer (84.6%), in a palliative (n = 6, 46.2%), adjuvant (n = 5, 38.5%), or maintenance setting (n = 2, 15.4%). Six patients (46.2%) received combination therapy with two ICIs. The median time from ICI initiation to hepatitis onset was 91 days (IQR 54–112), and patients received a median of four injections (range 2–8). Liver biopsy was performed in 10 patients (76.9%) (Table S1).

**Table 1:** Characteristics and clinical parameters of patients with severe ICI-induced hepatitis requiring plasma exchange.

Characteristics	Severe CHILI requiring TPE n = 13	Response or improvement n = 8	No response or worsening n = 5	*p* value
Age, years, median (Q25-Q75)	63 (57-67)	61.0 (±7.2)	64.2 (±8.4)	0.48
Gender, n (%)				0.999
Male	7 (53.8)	4 (50)	3 (60)	
Chronic liver disease, n (%)	3 (23.1)	1 (12.5)	2 (40)	0.51
Cancer, n (%)				0.235
Lung	3 (15.4)	2 (25)	1 (20)	
Melanoma	3 (15.4)	2 (25)	1 (20)	
Hepatocellular carcinoma	2 (15.4)	0	2 (40)	
Colorectal	1 (7.7)	0	1 (20)	
Kidney	2 (7.7)	2 (25)	0	
Breast	1 (7.7)	1 (12.5)	0	
ENT	1 (7.7)	1 (12.5)	0	
Liver metastases, n (%)	2 (15.4)	2	0	0.487
ICI indication				
Palliative	6 (46.2)	4 (50)	2 (40)	
Adjuvant	5 (38.5)	4 (50)	1 (20)	
Maintenance	2 (15.4)	0	2 (40)	
ICI regimen, n (%)				
Nivolumab-ipilimumab	4 (30.8)	3 (37.5)	1 (20)	
Durvalumab-tremelimumab	2 (15.4)	0	2 (40)	
Pembrolizumab	5 (38.5)	3 (37.5)	2 (40)	
Atezolizumab	1 (7.7)	1 (12.5)	0	
Nivolumab	1 (7.7)	1 (12.5)	0	
Biological assessment at CHILI diagnosis, mean (SD)				
AST (IU/L)	712.1 (765.2)	526.4 (281.82)	1,009.2 (1,199.4)	0.724
ALT (IU/L)	830.9 (1,076.9)	602.9 (335.2)	1,195.6 (1,735.6)	>0.999
ALP (IU/L)	483.2 (450.1)	484.3 (455.7)	481.6 (494.4)	0.833
GGT (IU/L)	693.9 (780.1)	829.5 (933.8)	476.8 (451.8)	0.943
Total bilirubin (μmol/L)	85.3 (92.1)	86.0 (116.7)	84.4 (53.8)	0.416
Conjugated bilirubin (μmol/L)	64.6 (63.3)	58.9 (75.7)	74.5 (40.8)	0.412
Prothrombin rate (%)	78.8 (27.6)	93.1 (20.11)	58.6 (24)	0.057
Creatinine (μmol/L)	76.5 (23.0)	84.1 (24.6)	63.0 (13.5)	0.151
Eosinophils (/mm^3^)	154.6 (150.8)	118.33 (±93.26)	209.0 (233.9)	0.409
Lymphocytes (/mm^3^)	1,130.2 (1,085.9)	760.9 (657.1)	1,684.3 (1,461.5)	0.204
Liver biopsy, n (%)	10 (76.9)	7 (87.5)	3 (60)	0.51
CHILI pattern, n (%)				0.478
Hepatocellular	10 (76.9)	7 (87.5)	3 (60)	
Mixed	2 (15.4)	1 (12.5)	1 (20)	
Cholestatic	1 (7.7)	0	1 (20)	
Time from ICI initiation to hepatitis onset, days, median (Q1-Q3)	91 (54-112)	111.62 (±77.8)	78.0 (±38.4)	0.393
CHILI treatment				
Steroids 1 mg/kg	10 (76.9)	7 (87.5)	3 (60)	
Steroids 2 mg/kg	3 (23.1)	1 (12.5)	2 (40)	
Escalation of steroids	7 (53.8)	6 (75)	1 (20)	
2^nd^ line treatment: MMF	12 (92.3)	8 (100)	4 (80)	
3^rd^ line treatment: tacrolimus	7 (53.8)	5 (62.5)	2 (40)	
UDCA	2 (15.4)	1 (12.5)	1 (20)	
Encephalopathy during hospitalization	5 (38.5)	1 (12.5)	4 (80)	**0.032**
Time from hepatitis to 2^nd^ line treatment, median (Q1-Q3)	15 (10-24.6)	15.8 (±12.8)	24.3 (±11.9)	0.295
Time from hepatitis to 3^rd^ line treatment, median (Q1-Q3)	28 (20-31)	30.2 (±10.1)	15.3 (±12.1)	0.091
Plasmapheresis (TPE)				
Filtration	11 (84.6)	6 (75)	5 (100)	0.487
Centrifugation	2 (15.4)	2 (25)	0	
Number of sessions, mean (SD)	4.3 (1.7)	4.0 (±1.2)	4.8 (±2.4)	0.649
Peak biology				
Total bilirubin (μmol/L)	361.8 (160.6)	301.1 (±151.3)	458.8 (±134.8)	0.084
ALT (IU/L)	1,423.8 (1,194.7)	1,593.3 (±1,114)	1,152.6 (±1,400.2)	0.222
ALP (IU/L)	638.1 (533.9)	705.8 (±572.2)	529.8 (±508.1)	0.586
Prothrombin rate (%)	45.8 (25.8)	55.0 (±22.8)	30.6 (±24.4)	0.095
Evolution during management, median (Q1-Q3)				
MELD score at CHILI diagnosis	15 (7-18.5)	11.57 (±5.5)	16.2 (±6.0)	0.197
MELD score before TPE	21 (19-27)	20.9 (±5.8)	25.2 (±9.9)	0.335
MELD score end of TPE	19 (14-24)	15.9 (±4.4)	27.4 (±9.6)	**0.013**
MELD score 7days after TPE	18 (14-21)	16.0 (±4.1)	24.7 (±9.5)	0.086
Length of follow up, days, median (Q1-Q3) mean (±SD)	136 (62-306)	326.9 (±254.9)	82.0 (±102.0)	**0.019**
Length of hospitalization, days, median (Q1-Q3) mean (±SD)	20 (13-51)	43.9 (±45)	32.0 (±16.7)	0.833
Normalization liver tests	5 (38.5)			
Alive at last follow-up	7 (53.8)			
Cause of death				
Liver failure	3 (23.1)			
Septic shock	1 (7.7)			
Cancer progression	2 (15.4)			

Data are presented as number (%), median (Q1-Q3) or mean (SD), unless otherwise specified. ALP, alkaline phosphatase; ALT, alanine aminotransferase; AST, aspartate aminotransferase; CHILI, checkpoint inhibitor-induced liver injury; GGT, gamma-glutamyltransferase; ICI, immune checkpoint inhibitor; IVIG, intravenous immunoglobulin; MELD, model for end-stage liver disease; MMF, mycophenolate mofetil; TPE, therapeutic plasma exchange; UDCA, ursodeoxycholic acid. Normality and hetereoskedasticity of continuous data were assessed with Shapiro-Wilk and Levene’s test respectively. Continuous outcomes were compared with unpaired Student’s *t* test, Welch t-test or Mann-Whitney *U* test according to data distribution. Discrete outcomes were compared with chi-squared or Fisher’s exact test accordingly.

At the time of CHILI, the median MELD score was 15 (IQR 7-18.5), the median peak of total bilirubin was 311 μmol/L (IQR 232-475), and the median peak of international normalized ratio was 2.34 (IQR 1.3-2.7). All hepatitis cases were grade 4 according to CTCAE and classified as severe for seven patients and moderate (or moderate severe) for six patients according to DILI Network and DILI Expert Working Group criteria. Hepatic encephalopathy occurred in five patients (38.5%) during hospitalization. Seven patients met the criteria for ALI or ALF at the time of TPE (Table S2).

**Fig. 1:**
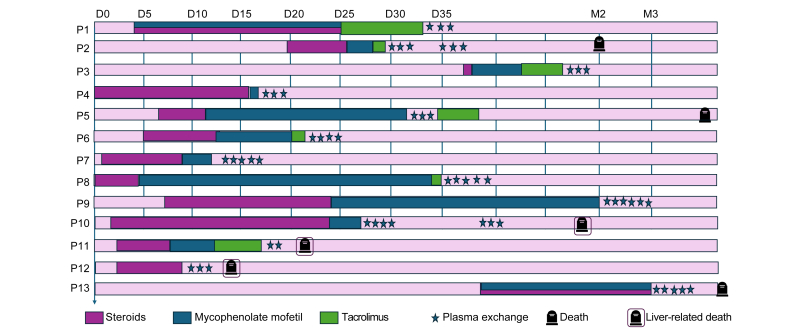
Treatment sequence in patients with immune checkpoint inhibitor-induced hepatitis. Each row (P1–P13) represents an individual patient. The horizontal axis indicates time from treatment initiation (D0: day 0; D5, D10, D15, D20, D25, D30, D35; M2: month 2; M3: month 3). Treatments are color-coded as follows: corticosteroids (pink), mycophenolate mofetil (blue), and tacrolimus (green). Plasma exchange sessions are indicated by blue star symbols. Black icons denote deaths from any cause, while black icons outlined in purple indicate liver-related deaths.

All patients received corticosteroid therapy as first-line treatment. In 12 patients, this was followed by second-line immunosuppressive therapy with mycophenolate mofetil (median delay: 15 days [IQR 10-24]), and in seven patients, third-line therapy with tacrolimus was initiated (median delay: 37 days [IQR 6-252]). One patient did not receive any second- or third-line immunosuppressive treatment. Two patients also received ursodeoxycholic acid (Fig. 1).

TPE was indicated for acute hepatitis in 11 patients, including seven with ALI or ALF, for a steroid-refractory cholestatic form in one patient, and for steroid-dependence in another patient, and was initiated at a median of 28 days (18–35) after the onset of hepatitis. At the time of TPE initiation, the median MELD score was 21 (10–40).

Patients underwent between two and eight PE sessions, by filtration (n = 11) or centrifugation (n = 2), with volumes ≥3,000 ml per session in most cases. Adverse events associated with PE included hypocalcemia, bleeding (n = 2), and thrombosis (n = 1). Intravenous immunoglobulins were co-administered in five patients during PE sessions. The median MELD score at the end of the PE sessions was 19 (range 8–39). After a mean follow-up of 260.9 days (±222.8), six patients (46.2%) had died. Among them, three deaths were due to liver failure, occurring 51, 21, and 15 days after hepatitis onset. The remaining deaths occurred 261 and 122 days later due to cancer progression, and 62 days later due to septic shock. Among the three patients who died from liver failure, two had underlying cirrhosis and were being treated for hepatocellular carcinoma. All patients who died from liver failure during hospitalization showed a mean increase in MELD score of +5.3 points, whereas the remaining patients experienced a mean MELD decrease of –4.5 points. Liver function tests improved or normalized in eight patients, and anticancer therapy without immunotherapy could be resumed in five of them. There was no notable difference between responders and non-responders, particularly regarding the number of TPE sessions (Table 1).

## Discussion

In this multicenter retrospective series, we report 13 cases of acute hepatitis induced by ICIs, all refractory to corticosteroids and at least one line of immunosuppressive therapy (except one patient), and treated with TPE. To our knowledge this is the largest cohort of patients treated with TPE for this indication. TPE resulted in improved or normalized liver function tests in eight patients (61.5%). Three patients, including two with underlying cirrhosis and hepatocellular carcinoma, died of ALF during hospitalization. No severe complications related to TPE were observed, suggesting the feasibility and safety of this approach in such high-risk patients.

Severe or fulminant forms of CHILI are rare, with an estimated incidence of 0.07% in a systematic review (3) and 0.02% (8/335 patients) in a recent study of patients with cirrhosis treated with nivolumab/ipilimumab for hepatocellular carcinoma.^15^ These patients with advanced cancer are usually ineligible for liver transplantation, thus emphasizing the urgent need for alternative rescue therapies.

TPE has already been used in other immune-related toxicities such as neurologic, cardiac, hematologic, or vasculitis syndromes, often improving clinical outcomes.^16^ In their review, Katsumoto *et al.* proposed TPE as an adjunctive strategy in the management of severe irAEs by enabling rapid clearance of pathogenic antibodies, pro-inflammatory cytokines and chemokines, and circulating therapeutic immunoglobulins, including ICIs.^16^ TPE is associated with an increase in regulatory T cell frequency, along with alterations in the number and activation status of both B and T lymphocytes.^17^ Given their high molecular weight, long half-life, and low extravascular distribution volume, ICIs are particularly susceptible to extracorporeal removal. The efficacy of TPE may be most effective when initiated early, ideally before irreversible liver injury has occurred.^16^ Although alternative third-line therapies have been reported, they remain unvalidated, and TPE should not be viewed as the sole therapeutic option.

Some expert teams also propose TPE in cases of fulminant hepatitis regardless of etiology, although its benefit on survival remains debated.^18^^,^^19^ In our series, patients who showed a decrease in MELD score between the beginning and end of TPE did not die from hepatic failure, whereas two patients with low initial MELD scores, cholestatic hepatitis, and corticosteroid resistance – yet no significant prothrombin time decrease – experienced limited benefit from TPE. Non-responders more frequently presented with hepatic encephalopathy during hospitalization and had a higher MELD score at the end of TPE. These observations raise questions about appropriate patient selection and the timing of intervention.

Adverse events associated with TPE occur in 2–3% of cases, with most being mild or moderate, and only 0.1% classified as severe.^20^ The prompt correction of liver function observed with TPE may represent a rationale for its use, especially when it could reduce the need for prolonged exposure to immunosuppressive agents such as tacrolimus in patients with underlying malignancies. Although cost and availability may be limiting factors, it must be weighed against the potential benefits, including reduced hospital stays and improved quality of life.

Importantly, several patients in our cohort were able to resume oncologic therapy following hepatitis resolution – a major goal in this context.^21^ In addition, long-term tumor responses have been reported even after early ICI discontinuation due to irAEs, particularly in patients who experienced high-grade immune toxicity.^22^ These encouraging results must be tempered by the retrospective nature of the study, the limited sample size, the absence of a control group, the physician-driven decision to initiate TPE, the heterogeneity in primary cancer types, patterns of liver injury, and the presence or absence of pre-existing liver disease. However, given the high mortality of severe ICI-induced hepatitis in patients ineligible for liver transplantation, our findings suggest that TPE may represent a promising therapeutic alternative. The role of early TPE implementation following corticosteroid and immunosuppressive treatment failure warrants further prospective investigation.

## Abbreviations

ALF, acute liver failure; ALI, acute liver injury; CHILI, checkpoint inhibitor–induced liver injury; ICI, immune checkpoint inhibitor; irAE, immune-related adverse event; MELD, model for end-stage liver disease; TPE, therapeutic plasma exchange.

## Authors’ contributions

LM, EDM, CM: study conception and design. LM, CM, EDM, AS, LH, OM, MK, CC, MF, FV, MR: data acquisition. LM: data analysis and interpretation. LM, CM, EDM: manuscript preparation and drafting. LM: statistical data analysis. All authors: manuscript reviewing. All authors have approved the final manuscript submitted.

## Data availability

The data that support the findings of this study are available on request from the corresponding author.

## Financial support

No financial support received to produce this manuscript.

## Conflict of interest

The authors declare no conflict of interest related to this work.
