# Supplementary material for: Plasma exchange as potential treatment of severe immune checkpoint inhibitor-induced hepatitis
Source: JHEP Rep. 2025 Nov 20;8(3):101684. doi: 10.1016/j.jhepr.2025.101684 (PMC12873720; doi:10.1016/j.jhepr.2025.101684)
Supplement: Multimedia component 1 [file mmc1.pdf]

Plasma exchange as potential treatment of severe immune checkpoint inhibitor-induced hepatitis

Lucy Meunier, Clement Monet, Antonio Saviano, Marwin Farrugia, François Villeret, Fanny Lebossé, Marion Khaldi, Olivier Moranne, Christine Chambon, Philippe Ichai, Astrid Laurent-Bellue, Ariane Laparra, Rodolphe Anty, Simona Tripon, Mialy Randrianarisoa, Alexandre Maria, Lina Hountondji, Eleonora De Martin

Table of contents

Table S1...2

Table S2...3

Table S1 : Histological description of liver biopsies

Pt	LB before steroids (Y/N)	Fibrosis (Metavir)	Inflammation			Interface hepatitis	Lobular necrosis	Granulomas	Bile duct injury	Cholestasis	Responder (Y/N)
			Site	Intensity	Characteristics						
1	No	F0	Portal and lobular	Moderate	Lymphocytes eosinophils, neutrophils and rare plasma cells	No	Confluent centrilobular (40%)	Micro-granulomas	No	No	Yes
2	Yes	F1	Lobular	Severe	Lymphocytes eosinophils, neutrophils and rare plasma cells	No	Confluent centrilobular and bridging	No	Ductular reaction	No	No
3	Yes	F1	Portal and lobular	Moderate	Lymphocytes eosinophils, neutrophils and plasma cells	Yes	No	No	No	No	Yes
4	No	F1	Portal and lobular	Moderate	Lymphocytes, eosinophils, neutrophils and rare plasma cells	Yes	Confluent centrilobular and periportal, and bridging (50%)	No	No	No	Yes
5	Yes	F0	Portal and lobular	Mild	Lymphocytes, rare eosinophils	No	Spotty lobular necrosis	Micro-granulomas	Lymphocytic cholangitis without ductular reaction	Yes (hepatocanalicular)	Yes
6	No	F0	Portal and lobular	Moderate	Lymphocytes, eosinophils	No	No	No	Lymphocytic cholangitis	Yes (hepatocellular)	Yes
7	Yes	F0	Portal and lobular	Moderate	Lymphocytes, rare eosinophils and plasma cells	Yes	No	No	Lymphocytic cholangitis without ductular reaction	Yes (hepatocanalicular)	No
8	No	F0	Portal and lobular	Moderate	Lymphocytes, histiocytes, neutrophils and eosinophils	No	Focal lobular necrosis	No	Lymphocytic cholangitis, degenerative changes	No	No
9	Yes	F1	Portal and lobular	Severe	Lymphocytes, histiocytes and rare plasma cells	Yes	Confluent centrilobular and bridging (20%)	No	Lymphocytic cholangitis, degenerative changes	Yes (hepatocellular)	No
10	No	F2	Portal and lobular	Moderate	Lymphocytes, histiocytes, rare neutrophils	Yes	Confluent centrilobular	No	Ductular reaction	Yes	No

Table S2 : Characteristics and clinical parameters of patients with severe ICI-induced hepatitis requiring plasma exchange, stratified by liver injury classification (ALI, ALF, and non-ALI/ALF).

Data are presented as number (%) or median (Q1-Q3). Abbreviations: CHILI, checkpoint inhibitor-induced liver injury; ICI, immune checkpoint inhibitor; AST, aspartate aminotransferase; ALT, alanine aminotransferase; ALP, alkaline phosphatase; GGT, gamma-glutamyl transferase; MELD, Model for End-stage Liver Disease; MMF, mycophenolate mofetil; UDCA, ursodeoxycholic acid; TPE, therapeutic plasma exchange; IVIG, intravenous immunoglobulin.

Normality and hetereoskedasticity of continuous data were assessed with Shapiro-Wilk and Levene's test respectively. Continuous outcomes were compared with unpaired Student t-test, Welch t-test or Mann-Whitney U test according to data distribution. Discrete outcomes were compared with chi-squared or Fisher's exact test accordingly.

**Characteristics**	**Hepatitis not meeting ALI/ALF criteria** n=6	**Acute liver injury (ALI)** n=2	**Acute liver failure (ALF)** n=5
**Age**, years, median (Q25-Q75)	62 (56-69)	63 (61-65)	63 (62-64)
**Gender**, *n (%)*			
Male	4 (66.7)	0 (0)	3 (60)
**Chronic liver disease,** *n (%)*	1 (16.7)	0 (0)	2 (40)
**Cancer** *n (%)*			
Lung	1 (16.7)	1 (50)	1 (20)
Melanoma	1 (16.7)	1 (50)	1 (20)
Hepatocellular carcinoma	0	0	2 (40)
Colorectal	0	0	1 (20)
Kidney	2 (33.3)	0	0
Breast	1 (16.7)	0	0
ENT	1 (16.7)	0	0
**Liver metastases,** *n (%)*	2 (33.3)	0	0
**ICI indication**			
Palliative	2 (33.3)	2 (100)	2 (40)
Adjuvant	4 (66.7)	0	1 (20)
Maintenance	0	0	2 (40)
**ICI regimen**, *n (%)*			
Nivolumab-Ipilimumab	2 (33.3)	1 (50)	1 (20)
Durvalumab-tremelimumab	0	0	2 (40)
Pembrolizumab	3 (50.0)	1 (50)	1 (20)
Atezolizumab	0	0	1 (20)
Nivolumab	1 (16.7)	0	0
**Biological assessment at CHILI diagnosis** median (Q25-Q75)			
AST (IU/l)	597.0 (336.3-747.5)	175.5 (153.8-197.3)	814.0 (384.0-1060.0)
ALT (IU/l)	526.0 (294.0-836.0)	309.0 (235.5-382.5)	906.0 (467.0-948.0)
ALP (IU/l)	825.0 (472.0-1209.0)	118.5 (104.8-132.3)	224.0 (161.0-233.0)
GGT (IU/l)	1376.0 (560.0-1794.0)	105.5 (76.8-134.3)	262.0 (132.0-297.0)
Total bilirubin (μmol/l)	50.0 (22.0-234.0)	5.0 (5.0-5.0)	73.0 (54.0-137.0)
Conjugated bilirubin (μmol/l)	45.0 (20.0-162.0)	3.5 (3.3-3.8)	50.0 (31.0-99.0)
Prothrombin rate (%)	100 (100-100)	70.0 (60.5-79.5)	50.0 (45.0-62.0)
Creatinine (μmol/l)	83.5 (69.5-97.5)	97.0 (90.0-104.0)	65.0 (61.0-67.0)
Eosinophils (/mm3)	80.0 (75.0-125.0)	195.0 (157.5-232.5)	76.0 (40.0-170.0)

Lymphocytes (/mm3)	1020.0 (895.0-1060.0)	2555.0 (2317.5-2792.5)	1670.0 (620.0-1897.0)
**Liver biopsy** *n (%)*	4 (66.7)	2 (100)	4 (80)
**CHILI pattern** *n (%)*			
Hepatocellular	4 (66.7)	2 (100)	4 (80)
Mixed	1 (16.7)	0	1 (20)
Cholestatic	1 (16.7)	0	0
**Time from ICI initiation to hepatitis onset** *days, median (Q1-Q3)*	84 (50.8-101.5)	132.0 (120.5-136.0)	55 (54-112)
**CHILI treatment**			
Steroids 1mg/kg	6 (100)	2 (100)	2 (40)
Steroids 2mg/kg	0	0	3 (60)
Escalation of steroids	4 (66.7)	1 (50)	2 (40)
2nd line treatment : MMF	6 (100)	2 (100)	4 (80)
3rd line treatment : tacrolimus	3 (50.0)	2 (100)	2 (40)
UDCA	2 (33.3)	0	0
**Encephalopathy during hospitalization**	0	0	5 (100)
**Time from hepatitis to 2nd line treatment,** *median (Q1-Q3)*	12.0 (10.3-21.3)	23.5 (13.8-33.3)	20.0 (15.3-23.5)
**Time from hepatitis to 3rd line treatment,** *median (Q1-Q3)*	21.0 (20.0-29.0) †	40.0 (36.0-44.0)	21.0 (14.0-28.0) ‡
**Plasmapheresis (TPE)**			
Filtration	5 (83.3)	1 (50)	5 (100)
Centrifugation	1 (16.7)	1 (50)	0
Number of sessions, median (Q1-Q3)	5.0 (4.3-5.0)	3.0 (3.0-3.0)	3.0 (3.0-6.0)
**Peak biology**			
Total bilirubin (μmol/l)	254.0 (217.8-420)	300.0 (243.5-383.0)	474.6 (466.0-550.0)
ALT (IU/l)	795.0 (678.0-1010.3)	1695.0 (991.0-2517.5)	906.0 (615.0-3307.0)
ALP (IU/l)	1156.0 (878.3-1343.0)	231.0 (206.5-232.0)	231.0 (150.0-245.0)
Prothrombin rate (%)	70.5 (66.8-75.8)	27.0 (17.0-30.0)	24.0 (23.0-27.0)
**Evolution during management** *median (Q1-Q3)*			
MELD score at CHILI diagnosis	10 (7-17)	11 (9-12)	18 (16-20)
MELD score before TPE	19 (14-20)	27 (26-27)	27 (25-27)
MELD score end of TPE	14 (13-16)	21 (20-21)	26 (24-34)
MELD score 7days after TPE	14 (12-15) †	18 (17-20)	28 (24-30) ‡
**Length of follow up** *days, median (Q1-Q3)*	203.0 (138.3-294.8)	302.5 (216.8-388.3)	51.0 (21.0-62.0)
**Length of hospitalization** *days, median (Q1-Q3*	18.5 (11.0-43.4)	46.0 (28.0-64.0)	32.0 (18.0-46.0)
**Normalization liver tests**	2 (33.3)	2 (100)	1 (20)
**Alive at last follow-up**	4 (66.7)	2 (100)	1 (20)
**Cause of death**			
Liver failure	0		3 (75)
Septic shock	0		1 (25)
Cancer progression	2 (33.3)		0

‡ Data available for n = 2 patients.
† Data available for n = 4 patients.
